# Associations Between Social Determinants of Health and Adherence to Biologic Therapies in Patients with Severe Asthma: Real-World Evidence from a Large US Claims Analysis

**DOI:** 10.2147/JAA.S608891

**Published:** 2026-06-25

**Authors:** Justin Kwiatek, Arijita Deb, Guillaume Germain, François Laliberté, Malena Mahendran, Patrick Gravelle, Annalise Hilts, Rohit K Katial, Diego J Maselli

**Affiliations:** 1 US Medical Affairs, GSK, Collegeville, PA, USA; 2 US Real-World Evidence & Health Outcomes Research, GSK, Collegeville, PA, USA; 3 Groupe d’analyse SRI, Montreal, QC, Canada; 4 Department of Medicine, National Jewish Health and University of Colorado, Denver, CO, USA; 5 Department of Medicine, University of Texas Health at San Antonio, San Antonio, TX, USA

**Keywords:** biologic product, initiation, medication adherence, social determinants of health

## Abstract

**Purpose:**

This study describes factors associated with biologic initiation and adherence, and impacts of adherence on exacerbations among US patients with severe asthma.

**Patients and Methods:**

This retrospective cohort study defined two populations, biologic-eligible patients and biologic users, using the Komodo Research Database of healthcare claims (2016–2024). The biologic-eligible cohort was defined as patients with severe asthma who had ≥2 exacerbations within 12 months, after the severe asthma index date. Severe asthma was defined as ≥3 month medium-to-high-dose inhaled corticosteroid use with ≥1 overlapping supply with controller therapy. The severe asthma index date was the first day of overlapping treatment supply. Biologic users were defined as patients with ≥2 dispensings of the same biologic and ≥2 asthma diagnoses in the year prior to biologic initiation. In the biologic-eligible cohort, biologic initiation was evaluated and compared between less- and more-favored social determinants of health (SDOH) areas (based on census-level quartiles). Adherence (assessed based on refills and treatment gaps) and exacerbations were assessed among biologic users in the 12 months following biologic initiation and compared between less- and more-favored SDOH areas.

**Results:**

Overall 29,652 patients had ≥2 years of follow-up in the biologic-eligible cohort. Of these, 11.3% (n=3362) initiated biologics. In total, 16,336 patients were included in the biologic-user cohort, among whom 62.6% were adherent, 12.1% partially adherent, 3.1% minimally adherent, and 22.3% discontinued treatment. Biologic initiation and adherence were lower in less- versus more-favored SDOH areas. Compared with adherent patients, those who were partially/minimally adherent or discontinued treatment in the 12 months following biologic initiation had significantly more exacerbations post-landmark period (+18% overall and +28% hospitalization-defined exacerbations).

**Conclusion:**

Disparities in biologic initiation and adherence exist between severe asthma populations with different characteristics and SDOH. Biologic adherence is associated with fewer exacerbations compared with partial adherence, minimal adherence, and treatment discontinuation.

## Plain Language Summary

Patients with severe asthma often experience flare-ups (exacerbations) and symptoms that cannot be controlled with treatments such as inhaled steroids and bronchodilators. Injectable biologic medicines that target the type of inflammation experienced by certain patients with asthma are now available. Biologics can reduce asthma flare-ups and improve safety compared with long-term oral steroids, if taken regularly.

Relatively few patients start (ie, initiate) biologic treatment. Of those patients who do, the number who keep taking their biologic regularly (ie, adhere to treatment) is low. Lack of biologic initiation and poor adherence can increase the risk of exacerbations for patients with severe asthma. Social determinants of health (SDOH; eg, education, race/ethnicity) can impact initiation and adherence.

Using data from the Komodo Research Database (2016–2024), this study assessed factors associated with biologic initiation and adherence and the impact of adherence on exacerbations among US patients with severe asthma. Among patients with 2 years of follow-up who were eligible for biologic therapy, only 11% initiated biologics. Biologic initiation and adherence were lower in less- versus more-favored areas, according to factors such as lack of high school diploma and racial or ethnic or minority status. Per year, patients who were not/less adherent to biologic therapy experienced 18% more exacerbations overall versus adherent patients.

These results highlight the need to consider the impact of SDOH factors when developing strategies to support patients initiating biologics so that they can have optimal adherence and hence better asthma outcomes.

## Introduction

Among the asthma population in the United States (US), approximately 14% have a severe phenotype^1^ that is predominantly driven by type 2 inflammation.^2^,^3^ Inadequate control of underlying inflammation in individuals with severe asthma may result in an increased risk of worsening symptoms and asthma exacerbations,^3^,^4^ potentially leading to airway remodeling and worse disease progression.^5^

Patients with severe asthma may require combination high-dose inhaled corticosteroid (ICS)/long-acting β_2_-agonist (LABA) treatment or biologic therapy to maintain symptom control and prevent exacerbations.^2^ The Global Initiative for Asthma (GINA) recommends considering a biologic as an add-on treatment for patients with uncontrolled asthma despite taking at least high-dose ICS-LABA and who have allergic or eosinophilic biomarkers (eg, blood eosinophil count >150 cell/µL) or need maintenance oral corticosteroids (OCS).^2^ Approved biologics targeting immunoglobulin E (omalizumab), interleukin(IL)-5 (mepolizumab, reslizumab, depemokimab), the IL-5 receptor (benralizumab), IL-4/IL-13 (dupilumab), or thymic stromal lymphopoietin (TSLP; tezepelumab) have been associated with improvements in exacerbation rates, asthma symptoms, and lung function, as well as reduced reliance on rescue medication and glucocorticoids in adults with moderate-to-severe or uncontrolled severe asthma.^6–14^

Initiation of and adherence to any medications are closely linked with patient outcomes.^15–18^ While biologic uptake for severe asthma has increased since 2015 in line with increasing availability, biologic initiation remains relatively low overall, particularly in patients with poorer social determinants of health (SDOH) such as lack of access to a respiratory specialist, lower income, and lack of commercial insurance coverage.^15^,^19^,^20^ For patients who do initiate biologic treatment, adherence is crucial given that non-adherence may result in inconsistent control of inflammation, leaving patients vulnerable to further exacerbations and disease progression.^2^,^5^,^20–23^ Factors associated with lower adherence include Black/African American race, Hispanic/Latino ethnicity, lower education level, Medicare or Medicaid medical insurance, lack of access to a specialist, and at-home administration.^20^,^24^ Approximately 10–13% of people receiving a biologic for severe asthma in the US between 2015 and 2022 discontinued treatment,^15^,^16^ despite a documented increased risk of poor outcomes with discontinuing versus continuing biologic therapy;^25–27^ discontinuation of biologics has been associated with higher baseline blood eosinophil count and exacerbation rate, poorer lung function, and greater healthcare resource utilization compared with continuing treatment.^15^,^16^,^20^,^24^ A recent study conducted in Japan highlighted the impact of adherence on patient outcomes, showing that adherence was associated with better clinical outcomes and lower healthcare utilization compared with reduced adherence.^28^ However, there is currently an evidence gap surrounding the factors impacting rates of biologic initiation and studies to date that have assessed adherence have been limited in size.^15^,^20^,^24^,^29–31^ An understanding of the factors contributing to biologic initiation and adherence is essential in supporting patients to achieve optimal treatment outcomes.

This study investigated the impact of patient characteristics and SDOH on biologic initiation among patients eligible for biologic treatment. Additionally, adherence and its impact on exacerbation rates was assessed among patients who initiated biologic treatment.

## Methods

### Study Design and Population

This retrospective cohort study utilized administrative claims data from the Komodo Research Database. This US-wide healthcare claims database is sourced from a variety of payers and healthcare organizations including commercial, Medicare and Medicaid insurers, and was chosen due to its broad patient coverage, which at the time of the study, included over 65 billion de-identified clinical, pharmacy, and laboratory encounters for more than 320 million patients enrolled in a health care plan in the US from 2016. Two patient cohorts were identified between January 1, 2016 and June 30, 2024: patients with severe asthma who were eligible for biologic treatment (biologic-eligible cohort) and patients who had initiated biologic treatment (biologic-user cohort).

Severe asthma was defined as patients with ≥3 months’ use of medium- or high-dose ICS with ≥1 overlapping day of supply with controller therapy (ie, ICS, LABA, and long-acting muscarinic antagonist [LAMA] containing therapies, including ICS/LABA, LAMA/LABA, and both single- and multiple-inhaler triple therapy), with the severe asthma index date defined as the first overlapping date of treatment supply. Patients with severe asthma who experienced ≥2 exacerbations in the year following severe asthma index were included in the biologic-eligible cohort, with the biologic-eligible index date defined as the date of the second exacerbation. These definitions generally align with the inclusion criteria for the pivotal trials for biologics indicated for asthma,^8^,^11^,^12^,^14^ and were used to identify patients who were suitable candidates for biologic treatment. Asthma exacerbations were defined by inpatient (IP)/emergency room (ER) visits or systemic corticosteroid (SCS) use. IP-/ER-defined exacerbations required an IP or ER visit (resulting in an IP visit within +1 day) where asthma was the primary diagnosis; SCS-defined exacerbations required an ER or outpatient visit where asthma was the primary diagnosis with a claim for 2–28 days’ supply of SCS within ±5 days. Patients were also required to be aged ≥12 years at the biologic-eligible index date and to have ≥12 months’ continuous insurance eligibility prior to the biologic-eligible index date. The biologic-eligible baseline period was defined as the 12-month period prior to the biologic-eligible index date, while the biologic-eligible follow-up period spanned from the biologic-eligible index date to the end of continuous insurance eligibility, end of data availability, or death, whichever came first (Supplemental Figure 1A).

The biologic-user cohort comprised patients who had ≥2 dispensings or administrations of the same biologic (benralizumab, dupilumab, mepolizumab, omalizumab, reslizumab, or tezepelumab), with the biologic-user index date defined as the date of first dispensing or administration. Patients in the biologic-user cohort also had ≥12 months' continuous insurance eligibility before and after the biologic-user index date, ≥2 asthma diagnoses on separate dates in the 12 months before the biologic-user index date (identified using International Classification of Diseases, Tenth Revision, Clinical Modification diagnostic codes [J45.3x–J45.5x, J45.9xx]) and were aged ≥12 years at the biologic-user index date. The 12 months before and after the biologic-user index date were defined as the biologic-user baseline and landmark periods, respectively. The landmark period was followed by the biologic-user follow-up period, which spanned from the end of the landmark period to the earliest occurrence of a switch to another biologic, end of continuous insurance eligibility, end of data availability, or death (Supplemental Figure 1B).

Patients in the biologic-eligible and biologic-user cohorts with ≥1 dispensing or administration for a biologic therapy any time prior to the biologic-eligible and biologic-user index dates, respectively, were excluded.

### Ethical Approval and Patient Consent

As this was a retrospective cohort study that analyzed de-identified health insurance claims data (ie, there was no direct patient contact or new data collection), it is not considered to be human subjects research under the US. Department of Health and Human Services federal regulation 45 CFR part 46. Informed consent, ethics committee, or institutional review board approvals were not required. The study complied with all applicable laws, regulations and guidance regarding patient protection, including patient privacy, all data were de-identified, and all study reports contained only aggregate results.

### Endpoints and Assessments

Patient demographics, clinical characteristics, and SDOH were evaluated during the respective 12-month baseline periods in the biologic-eligible and biologic-user cohorts; in the latter group, the data were stratified by adherence to biologic therapy. Race and ethnicity assignments within the Komodo Research Database were made primarily through information that is self-reported by the patient.

### Endpoints Assessed in the Biologic-Eligible Cohort

The cumulative incidence of biologic initiation was evaluated in biologic-eligible patients during the follow-up period. Biologic initiation among biologic-eligible patients was mapped by geographic location at the 3-digit zone improvement plan (ZIP) code level and reported overall, by type of biologic initiated, ICS dose, select asthma-related comorbidities, individual-level SDOH (race/ethnicity, insurance type), and area-level SDOH variables. The area-level SDOH variables were derived from the US Centers for Disease Control (CDC) and Prevention PLACES data (complete descriptions can be found on the CDC website).^32^ The PLACES SDOH data capture estimated prevalence of nine key measures that influence health and well-being. Further details on the SDOH can be found in the Supplementary Methods 1.

### Endpoints Assessed in the Biologic-User Cohort

Adherence to biologic therapy was evaluated clinically in the biologic-user cohort during the landmark period and patients were classified as adherent, partially adherent, minimally adherent, or discontinued treatment based on the number of refills and treatment gaps per label recommendations. Adherent patients had >50% refills and no treatment gaps of ≥2 doses (allowing for single doses to be missed occasionally); partially adherent patients had either ≤50% refills or a treatment gap of ≥2 doses; minimally adherent patients had ≤50% refills and a treatment gap of ≥2 doses; and discontinued patients had treatment gaps of ≥3 doses without biologic resumption. Adherence was mapped by geographic location at the 3-digit ZIP code level and reported by area- and individual-level SDOH variables and type of biologic initiated. Overall IP-/ER-defined asthma exacerbations were assessed during the follow-up period among the biologic-user cohort, with patients grouped into two adherence categories: adherent and not/less adherent (including partially adherent, minimally adherent, and discontinued treatment categories).

### Data Analysis

The cumulative incidence of biologic initiation among biologic-eligible patients was reported as the number and proportion of patients initiating a biologic during the follow-up period for area- and individual-level SDOH variables and at 1 year and 2 years following the biologic-eligible index date for overall incidence, type of biologic, ICS dose, and select asthma comorbidities.

The number and proportion of biologic users classified as adherent to biologic therapy during the landmark period were reported among patients living in less- versus more-favored SDOH areas (ie, 4th vs 1st quartiles, respectively, from the distribution of SDOH percentages at the 3-digit ZIP code level).

Geospatial mapping techniques (ie, bivariate choropleth mapping) were used to map biologic initiation among biologic-eligible patients and biologic adherence among the biologic-user cohort at the 3-digit ZIP code level.

For the biologic-user cohort, baseline characteristics were weighted between adherent and not/less adherent cohorts using the inverse probability of treatment weighting (IPTW) approach. Rates of asthma exacerbations were calculated as the number of events per person-year (PPY) and compared between weighted adherent versus not/less adherent cohorts using Poisson regression models with robust standard errors to estimate 95% confidence intervals (CIs) and p-values.

Proportion of days covered (PDC) was assessed for each patient in the biologic-user cohort, and was calculated as the sum of days of supply for each dispensing or administration of a biologic agent during the landmark period (after adjusting for overlapping dispensings), divided by the landmark period duration, and was presented stratified by adherence category.

## Results

### Patient Population

A total of 63,274 biologic-eligible patients and 16,336 biologic users were identified between January 1, 2016 and June 30, 2024.

### Characteristics of the Biologic-Eligible Cohort

The biologic-eligible cohort was mainly female (70.5%), had a mean (standard deviation [SD]) age of 49.3 (16.8) years, and over half of patients had commercial insurance (57.9%). At the biologic-eligible index date, 77.2% of patients were receiving high-dose ICS, and the mean (SD) number of exacerbations during baseline was 2.7 (1.1).

### Biologic Initiation Among Biologic-Eligible Cohort

**Figure 1:**
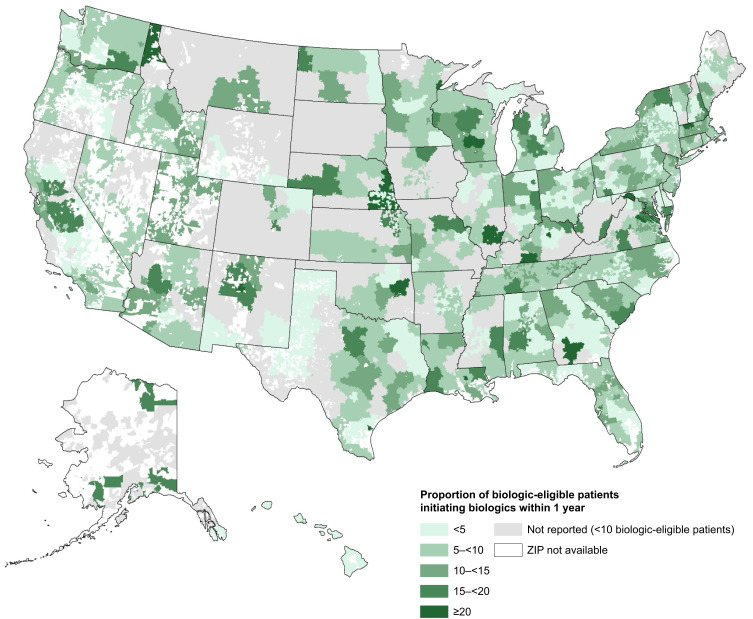
Geographic distribution of biologic initiation among biologic-eligible patients with severe asthma.

**Note**: Proportion of biologic initiators was calculated as the number of patients initiating a biologic divided by the total number of biologic-eligible patients (N=63,274) with ≥1 year of follow-up post-index.

**Abbreviation**: ZIP, zone improvement plan.

Among regions with at least 10 biologic-eligible patients, geospatial mapping identified that regions with biologic initiation ≥5% within 1 year of the biologic-eligible index date were most prevalent in the Northeast and Midwest, compared with those in the South and West (Figure 1).

Among 63,274 biologic-eligible patients, 29,652 had at least 2 years of follow-up. Among these patients, 3362 (11.3%) had initiated a biologic during follow-up, with dupilumab (3.4%) and benralizumab (2.6%) most frequently initiated (Table 1). Biologic initiation was more common among patients who were receiving high-dose ICS (12.5%) versus medium-dose ICS (7.0%). A higher proportion of patients with select asthma-related comorbidities initiated a biologic (ranging from 11.9% to 44.7%) versus those without comorbidities (9.7%). A larger proportion of patients with a respiratory specialty provider at the biologic-eligible index date initiated a biologic (18.3%) compared with those with a primary care provider (8.2%). Among those with a respiratory specialist provider, a slightly larger proportion of patients with an allergist provider initiated a biologic (21.0%) than those with a pulmonary specialist provider (16.7%). Regarding race/ethnicity status, Black/African American patients initiated biologic treatment (6.9%) less frequently compared with other groups (White: 8.0%; Hispanic/Latino: 7.1%; Asian or Pacific Islander: 8.3%). A higher proportion of biologic-eligible patients with commercial insurance initiated biologic treatment (9.4%) compared with those having Medicaid or Medicare insurance (6.4% and 6.1%, respectively).

**Table 1:** Cumulative Incidence of Biologic Initiation Among Biologic-Eligible Patients with Severe Asthma, Stratified by Type of Biologic, ICS Dose, Select Comorbidities, Race/Ethnicity, and Insurance Plan Type

Biologic Initiation*	Patients with Minimum Follow-Up^†^	
	1 Year Post-Index	2 Years Post-Index
**Biologic-eligible population, N**	43,630	29,652
Any biologic initiation^‡^, n (%)	3521 (8.1)	3362 (11.3)
Dupilumab	1042 (2.4)	1001 (3.4)
Benralizumab	813 (1.9)	767 (2.6)
Mepolizumab	755 (1.7)	746 (2.5)
Omalizumab	751 (1.7)	752 (2.5)
Tezepelumab	142 (0.3)	79 (0.3)
Reslizumab	20 (0.0)	19 (0.1)
**Severe asthma treatment**		
High-dose ICS, N	34,178	23,418
Biologic initiators, n (%)	3038 (8.9)	2927 (12.5)
Medium-dose ICS, N	9452	6234
Biologic initiators, n (%)	483 (5.1)	435 (7.0)
**Severe asthma with select comorbidities**^§^		
Severe asthma only, N	28,598	19,495
Biologic initiators, n (%)	1916 (6.7)	1882 (9.7)
With COPD, N	11,084	7436
Biologic initiators, n (%)	952 (8.6)	884 (11.9)
With NP, N	1643	1197
Biologic initiators, n (%)	423 (25.7)	404 (33.8)
With CSU, AD, or EOE, N	3789	2522
Biologic initiators, n (%)	466 (12.3)	411 (16.3)
With EGPA, N	68	47
Biologic initiators, n (%)	25 (36.8)	21 (44.7)
Provider/prescriber specialty**		
Primary care, N	23,436	15,982
Biologic initiators, n (%)	1280 (5.5)	1306 (8.2)
Respiratory specialist, N	11,312	7758
Biologic initiators, n (%)	1582 (14.0)	1419 (18.3)
*Pulmo*n*ary specialist*^††^, N	7268	4824
Biologic initiators, n (%)	927 (12.8)	805 (16.7)
*Allergist*^††^, N	4183	3031
Biologic initiators, n (%)	682 (16.3)	637 (21.0)
*Other*, N	8882	5912
Biologic initiators, n (%)	659 (7.4)	637 (10.8)
**Race/ethnicity**		
White, N	21,737	
Biologic initiators, n (%)	1746 (8.0)	
Black/African American, N	6763	
Biologic initiators, n (%)	465 (6.9)	
Hispanic/Latino, N	4673	
Biologic initiators, n (%)	334 (7.1)	
Asian or Pacific Islander, N	996	
Biologic initiators, n (%)	83 (8.3)	
Other, N	965	
Biologic initiators, n (%)	87 (9.0)	
Unknown, N	8,491	
Biologic initiators, n (%)	806 (9.5)	
**Insurance plan type, n (%)**		
Commercial, N	25,139	
Biologic initiators, n (%)	2360 (9.4)	
Medicaid, N	10,527	
Biologic initiators, n (%)	674 (6.4)	
Medicare, N	7953	
Biologic initiators, n (%)	487 (6.1)	
Other, N	6	
Biologic initiators, n (%)	0 (0.0)	

**Notes**: *Proportion of biologic initiators was calculated as the number of patients initiating a biologic divided by the total number of biologic-eligible patients with the minimum follow-up post-index; ^†^Follow-up period spanned from the biologic-eligible index date up to the earliest of end of continuous insurance eligibility, end of data availability, or death; ^‡^Two patients initiated multiple biologics on the same date after their biologic-eligible index date, so the sum of patients initiating an individual biologic is larger than the number of patients initiating any biologic; ^§^Select comorbidities identified as claims with primary diagnoses for the select comorbidity of interest. A confirmed diagnosis for CSU was defined as ≥2 diagnoses of either idiopathic (L50.1), other (L50.8), or unspecified urticaria (L50.9) OR ≥1 diagnosis of idiopathic (L50.1), other (L50.8), or unspecified urticaria (L50.9) and ≥1 diagnosis of angioedema (T78.3), separated by at least 6 weeks but no more than 12 months, in either order. Diagnoses were evaluated during the baseline period, excluding the index date; **Evaluated using medical or pharmacy claims on the biologic-eligible index date. In the case of multiple claims on the same day, the following hierarchy was assigned: respiratory specialist>primary care>other. Other provider specialties included, but were not limited to, diagnostic radiology, emergency medicine, pathology and critical care medicine; ^††^Patients with visits for both a pulmonary specialist and an allergist were reflected in the counts of each sub-specialty. However, these patients only contributed a single count to the overall respiratory specialist patient count.

**Abbreviations**: AD, atopic dermatitis; COPD, chronic obstructive pulmonary disease; CSU, chronic spontaneous urticaria; EGPA, eosinophilic granulomatosis with polyangiitis; EOE, eosinophilic esophagitis; ICS, inhaled corticosteroid; NP, nasal polyps.

**Table 2:** Cumulative Incidence of Biologic Initiation Among Biologic-Eligible Patients with Severe Asthma, Stratified by Area-Level SDOH

SDOH*^†^	Patients Living in More-Favored Areas (1st SDOH Quartile) [A]	Patients Living in Less-Favored Areas (4th SDOH Quartile) [B]	Biologic Initiation Reduction [B]/[A]
**Patients living in areas stratified by the following census SDOH variables^‡§^:**			
Crowding among housing units, N	8477	12,818	
Biologic initiators, n (%)	706 (8.3)	1014 (7.9)	–5%
No high school diploma among adults aged ≥25 years, N	10,298	9808	
Biologic initiators, n (%)	936 (9.1)	662 (6.7)	–26%
Housing cost burden among households, N	6807	14,048	
Biologic initiators, n (%)	605 (8.9)	1026 (7.3)	–18%
No broadband internet subscription among households, N	18,532	4474	
Biologic initiators, n (%)	1604 (8.7)	355 (7.9)	–9%
Persons of racial or ethnic or minority status, N	5302	14,053	
Biologic initiators, n (%)	454 (8.6)	1067 (7.6)	–12%
Persons living below 150% of the poverty level, N	16,247	7697	
Biologic initiators, n (%)	1401 (8.6)	527 (6.8)	–21%
Single-parent households, N	7471	11,825	
Biologic initiators, n (%)	684 (9.2)	869 (7.3)	–21%
Unemployment among people ≥25 years in the labor force, N	6745	9975	
Biologic initiators, n (%)	598 (8.9)	730 (7.3)	–18%

**Notes**: *Patients were stratified by their SDOH being among the lowest or highest quartile from the distribution of SDOH percentages at the 3-digit ZIP code level; complete definitions for the SDOH can be found on the US CDC website;^32^ †Follow-up period spanned from the biologic-eligible index date up to the earliest of end of continuous insurance eligibility, end of data availability, or death; ^‡^Proportion of biologic initiators was calculated as the number of patients initiating a biologic divided by the total number of biologic-eligible patients with ≥1 year of follow-up post-index and SDOH below or above the national lower or upper quartile, respectively; ^§^13 patients missing ZIP code information were excluded.

**Abbreviations**: CDC, Centers for Disease Control and Prevention; SDOH, social determinants of health; ZIP, zone improvement plan.

Among biologic-eligible patients with ≥1 year of follow-up, biologic initiation was observed less frequently among patients living in less-favored versus more-favored areas for all area-level SDOH variables (Table 2). The largest disparity was observed for patients residing in areas where a high proportion of residents have no high school diploma aged ≥25 years (26% lower rate of biologic initiation in the 4th vs the 1st quartile), are living below 150% of the poverty level (21% lower in the 4th vs the 1st quartile), and living in single-parent households (21% lower in the 4th vs the 1st quartile).

**Table 3:** Demographics and Clinical Characteristics of Biologic Users, Stratified by Adherence to Biologic Therapy, in the Unweighted Analysis

Characteristics*	Total (N=16,336)	Adherent N=10,226 (62.6%)	Partially Adherent N=1972 (12.1%)	Minimally Adherent N=499 (3.1%)	Discontinuation N=3639 (22.3%)
Age, years, mean ±(SD)	–	50.0 ± 15.8	47.5 ± 16.4	47.6 ± 16.0	47.9 ± 16.8
Female, n (%)	–	6773 (66.2)	1328 (67.3)	359 (71.9)	2459 (67.6)
Region, n (%)	–				
South	5746	3449 (33.7)	764 (38.7)	203 (40.7)	1330 (36.5)
Northeast	4531	2944 (28.8)	486 (24.6)	139 (27.9)	962 (26.4)
Midwest	3921	2496 (24.4)	463 (23.5)	91 (18.2)	871 (23.9)
West	2135	1336 (13.1)	259 (13.1)	66 (13.2)	474 (13.0)
Unknown/Other	3	1 (0.0)	0 (0.0)	0 (0.0)	2 (0.1)
Insurance plan type, n (%)					
Commercial	11,132	7159 (70.0)	1334 (67.6)	310 (62.1)	2329 (64.0)
Medicaid	2618	1505 (14.7)	347 (17.6)	107 (21.4)	659 (18.1)
Medicare	2584	1561 (15.3)	290 (14.7)	82 (16.4)	651 (17.9)
Unknown	2	1 (0.0)	1 (0.1)	0 (0.0)	0 (0.0)
Race/ethnicity, n (%)					
White	8258	5361 (52.4)	875 (44.4)	221 (44.3)	1801 (49.5)
Black/African American	2136	1182 (11.6)	332 (16.8)	87 (17.4)	535 (14.7)
Hispanic/Latino	1468	864 (8.4)	208 (10.5)	56 (11.2)	340 (9.3)
Asian or Pacific Islander	392	249 (2.4)	47 (2.4)	16 (3.2)	80 (2.2)
Other	370	235 (2.3)	39 (2.0)	12 (2.4)	84 (2.3)
Unknown	3712	2335 (22.8)	471 (23.9)	107 (21.4)	799 (22.0)
Provider/prescriber specialty^†^, n (%)					
Respiratory specialist	9293	5915 (57.8)	1094 (55.5)	280 (56.1)	2004 (55.1)
Primary care	2505	1543 (15.1)	323 (16.4)	66 (13.2)	573 (15.7)
Other	4538	2768 (27.1)	555 (28.1)	153 (30.7)	1062 (29.2)
Biological administration location, n (%)					
Office	8885	5760 (56.3)	957 (48.5)	265 (53.1)	1903 (52.3)
At home	7451	4466 (43.7)	1015 (51.5)	234 (46.9)	1736 (47.7)
Severe asthma diagnosis^‡^, n (%)	11,487	7302 (71.4)	1348 (68.4)	358 (71.7)	2479 (68.1)
Asthma exacerbations, mean ±(SD)					
Overall	–	3.2 ± 1.5	3.2 ± 1.6	3.2 ± 1.6	3.1 ± 1.5
IP-/ER-defined	-	0.8 ± 1.3	1.0 ± 1.3	1.0 ± 1.4	1.0 ± 1.4
Proportion of days covered^§^, mean (IQR)	-	0.90 (0.84, 0.98)	0.69 (0.58, 0.80)	0.36 (0.28, 0.44)	0.35 (0.19, 0.50)

**Notes**: *Baseline characteristics presented using unweighted results and evaluated on the biologic-user index date; ^†^Evaluated using medical or pharmacy claims on the biologic-user index date. In the case of multiple claims on the same day, the following hierarchy was assigned: respiratory specialist>primary care>other. Other provider specialties included, but were not limited to, unspecified specialist, diagnostic radiology, emergency medicine, otolaryngology, and physical therapist; ^‡^Severe asthma diagnosis comprises primary or secondary diagnoses; ^§^Proportion of days covered was calculated for each patient as the sum of days of supply for each dispensing or administration of a biologic agent, after adjusting for overlapping dispensings, during the landmark period divided by the landmark period duration.

**Abbreviations**: ER, emergency room; IP, inpatient; IQR, interquartile range; SD, standard deviation.

### Characteristics of Biologic Users

Of 16,336 patients in the biologic-user cohort, 10,226 (62.6%), 1972 (12.1%), and 499 (3.1%) were classified as adherent, partially adherent, and minimally adherent, respectively, and 3639 (22.3%) had discontinued treatment. Biologic-user demographics and clinical characteristics during baseline or on the biologic-user index date in the unweighted analysis are presented in Table 3, stratified by adherence to biologic therapy. Mean age was 47.5–50.0 years, 66.2–71.9% were female, 68.1–71.7% had a primary or secondary diagnosis of severe asthma, and 62.1–70.0% had commercial insurance. The mean number of asthma exacerbations (any type) in the year prior to biologic initiation was 3.2 in all adherence categories. Baseline patient characteristics were generally similar between the adherent, partially adherent, minimally adherent, and discontinuation groups, although the mean age of patients in the adherent group (50.0 years) was approximately 2 years older than those in other groups (47.5–47.9 years). However, following IPTW, all patient demographics and clinical characteristics were well balanced (ie, standardized differences <10%; Supplemental Table 1).

### Adherence Among Biologic Users

**Figure 2:**
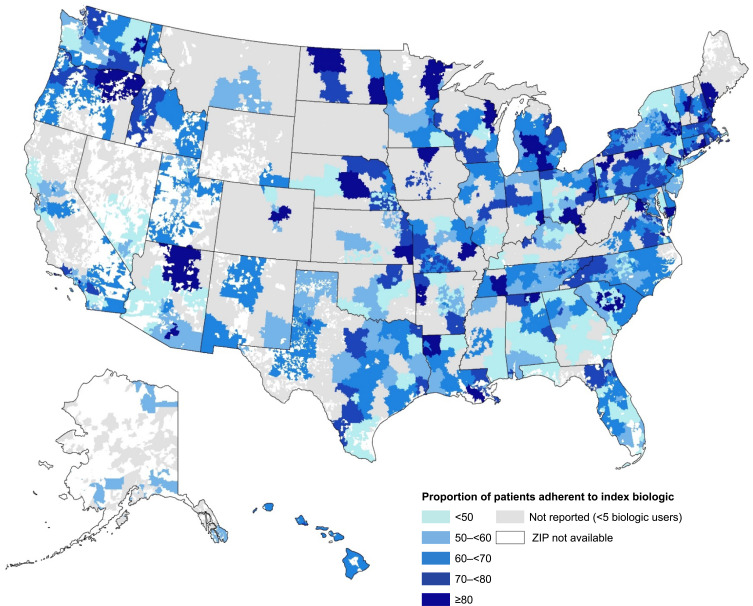
Geographic distribution of adherence to biologic therapy among biologic users.

**Note**: Adherence (among biologic users, n=16,336) was evaluated from the biologic-user index date to 12 months following this date. 13 patients were excluded because of missing ZIP code information.

**Abbreviation**: ZIP, zone improvement plan.

The geographic distribution of biologic users adherent to biologic therapy is shown in Figure 2. Adherence varied across and within states. States in which there were ≥50% adherent patients in a majority of ZIP code regions were concentrated in the Northeast, in particular Massachusetts, Connecticut, and Pennsylvania. In large geographical regions of the Midwest and West there were fewer than 5 patients in the biologic-user cohort and these regions were therefore not evaluated for proportion of adherent patients. States with regions having <50% adherent biologic users were most prevalent in the South, in particular Georgia, Alabama, and Florida.

**Table 4:** Adherence to Biologic Among Biologic Users, Stratified by Area-Level SDOH

SDOH*^†^	Patients Living in More-Favored Areas (1st SDOH Quartile) [A]	Patients Living in Less-Favored Areas (4th SDOH Quartile) [B]	Biologic Adherence Reduction [B]/[A]
**Patients living in areas stratified by the following census SDOH variables^‡§^:**			
Crowding among housing units, N	3350	4349	
Adherent patients, n (%)	2216 (66.1)	2596 (59.7)	–10%
No high school diploma among adults aged ≥25 years, N	4252	3083	
Adherent patients, n (%)	2809 (66.1)	1842 (59.7)	–10%
Housing cost burden among households, N	2848	4705	
Adherent patients, n (%)	1850 (65.0)	2831 (60.2)	–7%
No broadband internet subscription among households, N	7166	1698	
Adherent patients, n (%)	4569 (63.8)	1059 (62.4)	–2%
Persons of racial or ethnic or minority status, N	2075	4864	
Adherent patients, n (%)	1388 (66.9)	2901 (59.6)	–11%
Persons living below 150% of the poverty level, N	6432	2456	
Adherent patients, n (%)	4182 (65.0)	1481 (60.3)	–7%
Single-parent households, N	3048	4084	
Adherent patients, n (%)	1983 (65.1)	2441 (59.8)	–8%
Unemployment among people ≥25 years in the labor force, N	2733	3288	
Adherent patients, n (%)	1801 (65.9)	1983 (60.3)	–8%

**Notes**: *Patients were stratified by their SDOH being among the lowest or highest quartile from the distribution of SDOH percentages at the 3-digit ZIP code level; complete definitions for the SDOH can be found on the US CDC website;^32^ †Adherence was evaluated from the biologic-user index date to 12 months following this date; ^‡^Proportion of patients adherent to biologic therapy was calculated as the number of patients adherent to biologic therapy during the landmark period with SDOH meeting the defined threshold, divided by the total number of patients with SDOH meeting the defined threshold; ^§^1 patient missing ZIP code information was excluded.

**Abbreviations**: CDC, Centers for Disease Control and Prevention; SDOH, social determinants of health; ZIP, zone improvement plan.

Among biologic users, adherence to biologic therapy was observed less frequently among patients living in less-favored versus more-favored areas for all area-level SDOH variables (Table 4). The largest disparity was observed for patients residing in areas where a high proportion of residents are of racial or ethnic or minority status (11% lower adherence in the 4th vs the 1st quartile), have no high school diploma aged ≥25 years (10% lower in the 4th vs 1st quartile), and are experiencing crowding among housing units (10% lower in the 4th vs 1st quartile). Smaller disparities in adherence to biologic therapy among biologic users were observed compared with those in biologic initiation among biologic-eligible patients, between more and less-favored areas (Table 2 and Table 4). Among biologic users, a lower proportion of Black/African American patients were adherent (55.3%) compared with other groups (White: 64.9%; Hispanic/Latino: 58.9%; Asian or Pacific Islander: 63.5%). Patients with commercial insurance were more frequently adherent (64.3% of patients) compared with patients whose insurance was Medicaid or Medicare (57.5% and 60.4% of patients, respectively).

**Figure 3:**
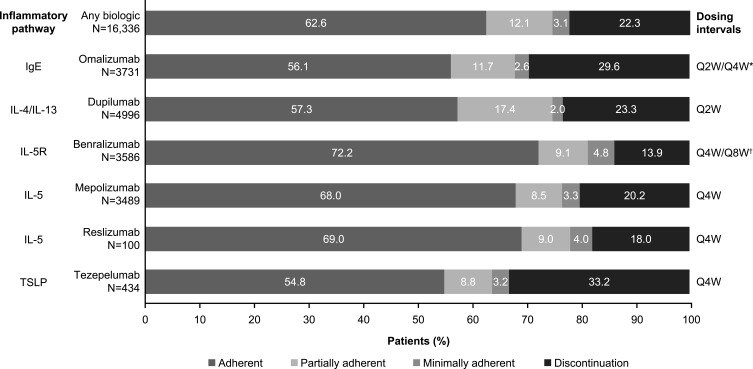
Adherence to biologic therapy among biologic users, stratified by biologic agent.

**Notes**: *Omalizumab dosed Q2W or Q4W, based on days of supply as a proxy for body weight and IgE level; ^†^Benralizumab dosed Q4W for the first three doses, then Q8W. Adherent patients had >50% refills and no treatment gaps of ≥2 doses, partially adherent patients had either ≤50% refills or a treatment gap of ≥2 doses, minimally adherent patients had ≤50% refills and a treatment gap of ≥2 doses, and discontinuation patients had treatment gaps of ≥3 doses without biologic resumption.

**Abbreviations**: IgE, immunoglobulin E; IL, interleukin; QxW, every x weeks; TSLP, thymic stromal lymphopoietin.

When stratified by biologic agent, the proportion of patients adherent to biologic therapy was highest among biologic users receiving benralizumab (72.2%), reslizumab (69.0%), or mepolizumab (68.0%). Discontinuation was highest for tezepelumab (33.2%) and omalizumab (29.6%) (Figure 3).

### Asthma Exacerbations Among Biologic Users

**Figure 4:**
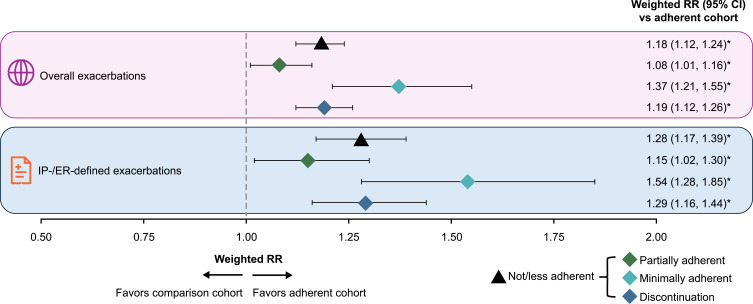
Rates of asthma exacerbation during follow-up among biologic users.

**Notes**: *p<0.05. Adherent patients had >50% refills and no treatment gaps of ≥2 doses, partially adherent patients had either ≤50% refills or a treatment gap of ≥2 doses, minimally adherent patients had ≤50% refills and a treatment gap of ≥2 doses, and discontinuation patients had treatment gaps of ≥3 doses without biologic resumption. Raw rates (PPY) for exacerbations by adherence group: adherent (n=10,226) 0.92 overall, 0.27 IP-/ER-defined; partially adherent (n=1972) 1.00 overall, 0.31 IP-/ER-defined; minimally adherent (n=499) 1.26 overall, 0.41 IP-/ER-defined; discontinued (n=3639) 1.10 overall, 0.35 IP-/ER-defined; and combined not/less adherent (n=6110) 1.09 overall, 0.34 IP-/ER-defined.

**Abbreviations**: CI, confidence interval; ER, emergency room; IP, inpatient; PPY, per patient-year; RR, rate ratio.

Patients categorized as not/less adherent to biologic therapy experienced significantly higher rates of exacerbations PPY versus adherent patients; specifically, this group had an 18% higher rate of exacerbations overall (rate ratio [95% CI]: 1.18 [1.12, 1.24], p<0.001) and a 28% higher rate of IP-/ER-defined exacerbations (rate ratio [95% CI]: 1.28 [1.17, 1.39], p<0.001) (Figure 4).

## Discussion

In this real-world, retrospective cohort study, biologic initiation was evaluated among 63,274 biologic-eligible patients, and adherence to biologic therapy and asthma exacerbations were evaluated among 16,336 biologic users.

Among biologic users, adherence was low across all groups with only around 60% of patients meeting the criteria for adherence; additionally, over 15% of patients were partially or minimally adherent and over 20% of patients discontinued treatment. These data highlight a consistent need for improved adherence in a substantial proportion of patients with severe asthma in the US. These findings were in line with those in the observational CHRONICLE study (including 2793 US patients with severe asthma) in 2018–2022,^20^ in which a 74% adherence rate was reported in Year 1 of biologic therapy. Notably, the CHRONICLE study defined adherence as ≥75% PDC,^20^ whereas the current study defined adherence as >50% refills and no treatment gaps of ≥2 doses and partial adherence as either ≤50% refills or a treatment gap of ≥2 doses. Despite the differing definitions, the corresponding mean PDC in the current study was 0.90 for adherent and 0.69 for partially adherent patients, suggesting that the clinical definitions used generally align with percentage-based approaches, although no standardized adherence definitions exist. Together, these data highlight a consistent need for improved adherence in over one-quarter of US patients with severe asthma.

Aside from patients who were adherent, partially adherent, and minimally adherent in the current study, just over one-fifth of biologic users discontinued their biologic in the year following initiation. These figures were similar to those from an analysis that combined real-world data from the CHRONICLE study in the US (2018–2020) and the International Severe Asthma Registry (ISAR) in 10 other countries (2015–2020), in which 79% of patients continued their first biologic for 6 months, 11% switched their biologic during follow-up, and 10% stopped their biologic during follow-up.^15^ Building on data from the CHRONICLE and ISAR studies, these results further confirm a relatively high rate of biologic discontinuation in US patients with severe asthma in routine clinical practice based on retrospective assessment of administrative claims data.^15^,^16^,^20^

Given the importance of biologic adherence for patients with severe asthma, further understanding of the factors driving non-adherence and discontinuation is important to ensure optimal patient outcomes. Patient demographic, disease-related, and medication-related factors previously associated with better adherence in patients with asthma include older age, commercial insurance, comorbid allergic rhinitis, chronic rhinosinusitis or atopic dermatitis, greater number of prescribed asthma medications, and access to specialist care.^16^,^20^,^24^,^31^ Consistent with previous findings, minimally adherent patients in the current study were of a lower age, a higher proportion insured by Medicaid, and a greater proportion were Black/African American compared with adherent patients.

Black race and Medicaid insurance have previously been associated with reduced initiation and adherence to biologic therapy for asthma.^29^ Similarly, in the current study, SDOH factors were associated with both biologic initiation and biologic adherence, with lower rates of both initiation and adherence among patients living in less- versus more-favored areas for all area-level SDOH variables explored. Less than 60% of biologic users were adherent to biologic therapy among those living in areas with the highest quartile for the following SDOH variables: people of racial or ethnic or minority status, adults aged ≥25 years without a high school diploma, or people living in crowded housing conditions. This analysis of SDOH variables associated with reduced biologic initiation and adherence reinforces the complex intersectionality of race, socioeconomic deprivation, insurance status, and health outcomes in the US as they relate to severe asthma. For example, compared with White patients, Black/African Americans patients are over-represented in the proportion of individuals with no health insurance or public health insurance (eg, Medicaid, Medicare),^33^ and in those with individual SDOH factors.^34–36^

Poor adherence to biologic therapy has clinical consequences including increased risk of poor asthma control and exacerbations.^37^ These data indicate that being not/less adherent to biologic therapy was associated with higher rates of asthma exacerbations, including 28% more IP-/ER-defined exacerbations compared with biologic-adherent patients. Prevention of asthma exacerbations is a key component of long-term asthma risk minimization, as each additional exacerbation can significantly decrease lung function.^5^,^38^ Addressing adherence to biologic therapy through all means, including reducing SDOH disparities, may improve long-term asthma control across the US, particularly among patients at greater risk for worse asthma-related outcomes. This may be particularly important in the states or regions with the highest burden of severe asthma exacerbations, such as Nevada and southern Florida, where the rate of IP-/ER-defined exacerbations is higher than the national average,^39^ but where biologic adherence rates were recorded as being as low as <50% in some areas.

While some patient demographic and clinical factors differed between adherence groups in the current study, some factors were generally similar. For example, the proportion of biologic users seeing a respiratory specialist was similar among adherent (58%) and minimally adherent (56%) patients, despite access to a specialist previously being associated with higher biologic adherence.^24^ Moreover, the median number of exacerbations in the year prior to biologic initiation was similar across all adherence groups (median 3.1–3.2 for overall exacerbations and 0.8–1.0 for IP-/ER-defined exacerbations), suggesting that prior exacerbations were not a major factor influencing biologic adherence. In a 2019 retrospective analysis of data from 85 patients with severe asthma, the most common patient-reported reason for biologic discontinuation was patient request (46.9% of respondents).^31^ As such, patient preference or other factors that are not recorded in healthcare insurance claims data may influence individual patients’ adherence behavior. For example, patients with severe asthma and their physicians have reported a preference for less frequent biologic administration (every 8 weeks [Q8W] vs every 4/2 weeks [Q4W/Q2W]).^22^ The current study results suggest that this previously reported preference for longer dosing intervals may be reflected in real-world adherence patterns, with adherence among biologic users highest (72.2%) for benralizumab (Q4W/Q8W), closely followed by reslizumab (69.0%; Q4W) and mepolizumab (68.0%; Q4W), then omalizumab (56.1%; Q2W/Q4W), tezepelumab (54.8%; Q4W), and dupilumab (57.3%; Q2W). Similar to these findings, the CHRONICLE study reported the highest median adherence, determined by PDC over the first 12 months of biologic therapy, with reslizumab (92%) and mepolizumab (89%), and the lowest with dupilumab (83%).^20^ Taken together, these findings suggest that adherence may be higher for biologics with longer dosing intervals. Biologics with alternative dosing schedules and extended half-lives may therefore be a therapeutic option, as these allow for less frequent administration. Examples include verekitug, a TSLP receptor antibody in Phase II development for severe asthma,^40^ and depemokimab, an ultra-long-acting biologic with enhanced IL-5 binding affinity, high potency, and an extended half-life (enabling twice-yearly dosing) which has demonstrated a reduction in annualized exacerbation rate versus placebo in patients with type 2 asthma characterized by blood eosinophil count in Phase III studies.^12^

The strengths of this study include its large sample size covering different regions across the US, sufficient follow-up time to determine biologic discontinuation, and analyses by biologic eligibility, biologic initiation, and for individual biologics. However, there are some limitations. This study may not be generalizable outside of the US setting due to differences in the structure and accessibility of healthcare services between countries. Assumptions were made regarding pharmacy data, particularly for biologics dispensed as multiple doses at once (eg, every second dose of dupilumab was assumed to be on time since it is dispensed as a 28-day supply), as well as the assumption that all dispensed biologics were taken by the patient. These assumptions could lead to misclassification of adherence, but it should be noted that this would likely result in an overestimation of adherence. Secondly, due to this study being claims-based, biomarker data such as blood eosinophil counts and fractional exhaled nitric oxide (FeNO) were not available. The exclusion of biomarker data from the biologic-eligibility criteria used for this study could be considered a limitation. However, it should be noted that in addition to allergic and eosinophilic asthma, some biologics are also indicated for OCS-dependent asthma. IPTW was used to adjust for differences in observed characteristics between cohorts. However, there was a possibility of residual confounding due to unobserved characteristics such as lung function, underlying clinical reasons, and other patient characteristics (eg, smoking status, FeNO, blood eosinophil counts, IgE level) that were not available in claims data. Thirdly, the timing of this study overlapped with the COVID-19 pandemic, so biologic initiation during this period may not be generalizable to other time periods given limited access to healthcare providers and resource utilization during this time. Thus, sensitivity analyses were conducted to assess the impact of COVID-19 on adherence and asthma-related outcomes. The analysis from the main study was replicated using only pre-COVID-19 data (up to February 29, 2020) to confirm the generalizability of the main study results. The findings from the COVID-19 sensitivity analysis were consistent with those from the full population (data not shown). Fourthly, SDOH variables were based on area codes rather than at a patient level, which limits the granularity of the data. Additionally, SDOH variables were evaluated during the baseline period or at the biologic-eligible or biologic-user index date. As such, changes in the pattern of biologic therapy utilization due to changes in these SDOH variables over the course of the study may not have been reflected. It should also be considered that as this study utilized claims data, it is not possible to know the indication that the biologic was prescribed for or the reasons (eg, costs, adverse events or lack of efficacy) that patients were non-adherent or discontinued their biologic. However, as the definition for biologic-eligible patients included the requirement for ≥2 exacerbations in the year prior to the biologic-eligible index date, it was likely that any subsequent biologic prescription would have been for asthma. The use of ER or IP admissions as a proxy for exacerbations may have been influenced by patient access to healthcare and potentially affected the interpretation of these outcomes. Finally, patients were required to have no biologic therapy in the 12 months prior to study entry. The goal was to limit the potential impact of prior biologic experience on adherence, but by doing so, patients who switched biologics were excluded, possibly accounting for the low number of patients receiving tezepelumab in this study.

## Conclusion

In conclusion, despite longstanding availability of biologics approved for severe asthma in the US, disparities in their initiation and adherence exist between patients with different SDOH variables. Dosing schedule also appears to be an important factor in adherence. Since non-adherence can reduce the clinical benefit of biologic therapy and put patients at risk of exacerbations and disease progression, these findings are relevant to patient management and should be taken into consideration when initiating and managing patients on biologic treatment. This large claims database study reveals challenges in initiation and adherence to biologics for patients with asthma, but more detailed, granular data (eg, utilizing electronic medical records) may help to better identify and predict trends in biologic use over time. This study also highlights the need for policies and strategies that take into consideration biologics with longer dosing intervals, addressing barriers related to insurance and SDOH variables, and engaging patients in education and shared decision-making to help optimize adherence and improve clinical outcomes for patients with severe asthma.

## Data Availability

Please refer to GSK weblink to access GSK’s data sharing policies and as applicable seek anonymized patient level data via the link https://www.gsk-studyregister.com/en/.

## Abbreviations

AD, atopic dermatitis; CDC, Centers for Disease Control and Prevention; CI, confidence interval; COPD, chronic obstructive pulmonary disease; CSU, chronic spontaneous urticaria; EGPA, eosinophilic granulomatosis with polyangiitis; EOE, eosinophilic esophagitis; ER, emergency room; ICS, inhaled corticosteroid; IgE, immunoglobulin E; IL, interleukin; IP, inpatient; IPTW, inverse probability of treatment weighting; IQR, interquartile range; NP, nasal polyps; PDC, proportion of days covered; PPY, per patient-year; QxW, every x weeks; RR, rate ratio; SCS, systemic corticosteroid; SD, standard deviation; SDOH, social determinants of health; TSLP, thymic stromal lymphopoietin; US, United States; ZIP, zone improvement plan.
